# The genetics of hyper IgE syndromes

**DOI:** 10.3389/fimmu.2025.1516068

**Published:** 2025-02-18

**Authors:** Randa AlYafie, Dinesh Velayutham, Nicholas van Panhuys, Puthen Veettil Jithesh

**Affiliations:** ^1^ College of Health and Life Sciences, Hamad bin Khalifa University, Doha, Qatar; ^2^ Laboratory of Immunoregulation, Research Department, Sidra Medicine, Doha, Qatar

**Keywords:** Mendelian disorders, primary immunodeficiency disorders, HIES, elevated serum IgE, immune signaling, genetic etiology, recurrent infections, atopic dermatitis

## Abstract

Hyper IgE syndromes (HIES) form a rare group of primary immunodeficiency disorders (PIDs) distinguished by persistent skin abscesses, dermatitis, allergies, and infections, in addition to their characteristic high serum IgE levels. Autosomal dominant (AD) and autosomal recessive (AR) genetic defects have been reported in HIES. From a clinical perspective, AD-HIES cases generally exhibit several non-immunologic features, including connective tissue, dental and skeletal abnormalities, whilst AR-HIES conditions have a higher incidence of neurologic complications and cutaneous viral infections. Genetic defects associated with HIES lead to impaired immune signaling, affecting pathways crucial for immune cell development, function, and immune response to pathogens/allergens. As a result, HIES patients are predisposed to recurrent bacterial and/or fungal infections, as well as atopic allergic responses. In many cases, the exact biological mechanisms responsible for the variations observed in the clinical phenotypes between the two inherited forms of HIES are still unclear. In this review, we describe the genetic basis of HIES with a distinction between the AR-HIES and AD-HIES forms, to better comprehend the different underlying molecular mechanisms, a distinction which is imperative for the accurate diagnosis, management, and development of targeted therapies for HIES patients.

## Introduction

Hyper IgE syndromes (HIES) are a category of primary immunodeficiency disorders (PIDs) broadly characterized by three major features: hyper-elevated serum IgE levels, enhanced susceptibility to infection, and eczema/atopic dermatitis, which together form the HIES symptomatic triad (1). Typically, eczema appears in early infancy coincident with bacterial infections, of which *Staphylococcus* infection is especially common, mostly in the respiratory system and skin. HIES-associated eczema manifests as “cold skin abscesses” which lack obvious indications of inflammation, such as redness and warmth. These abscesses can also develop in the joints (arthritis), sinuses (sinusitis), bones (osteomyelitis), bronchi (bronchitis), and lungs (resulting in pneumonia) (2). Dental, skeletal, and connective tissue abnormalities, as well as susceptibility to viral skin infections, are also common (3). The normal adult total serum IgE is commonly considered as below 140 UI/mL, though this range may vary based on ethnicity and age. In HIES, serum IgE levels often observed to be ten times greater than those seen at the top of the range for unaffected individuals (4).

## Genetic classification

HIES results from either spontaneous *de novo* genetic changes or can be inherited in either an AR or AD manner (5). The majority of AD-HIES cases currently diagnosed, have been linked to mutations in the *STAT3* gene, while the most common cause of AR-HIES is mutations in *DOCK8* (6). In 2006, Minegishi et al. identified the first genetic cause of HIES and described an autosomal recessive TYK2 deficiency in a single patient (7). This case was atypical, as subsequent reports of TYK2 deficiency did not consistently exhibit HIES. However, the documented impairment of IL-6 and IL-10 responses in this patient helped lead to the identification of autosomal dominant STAT3 deficiency in individuals with severe HIES (8), encouraging researchers to investigate additional JAK/STAT genes as potential contributors to autosomal dominant HIES.

**Figure 1:**
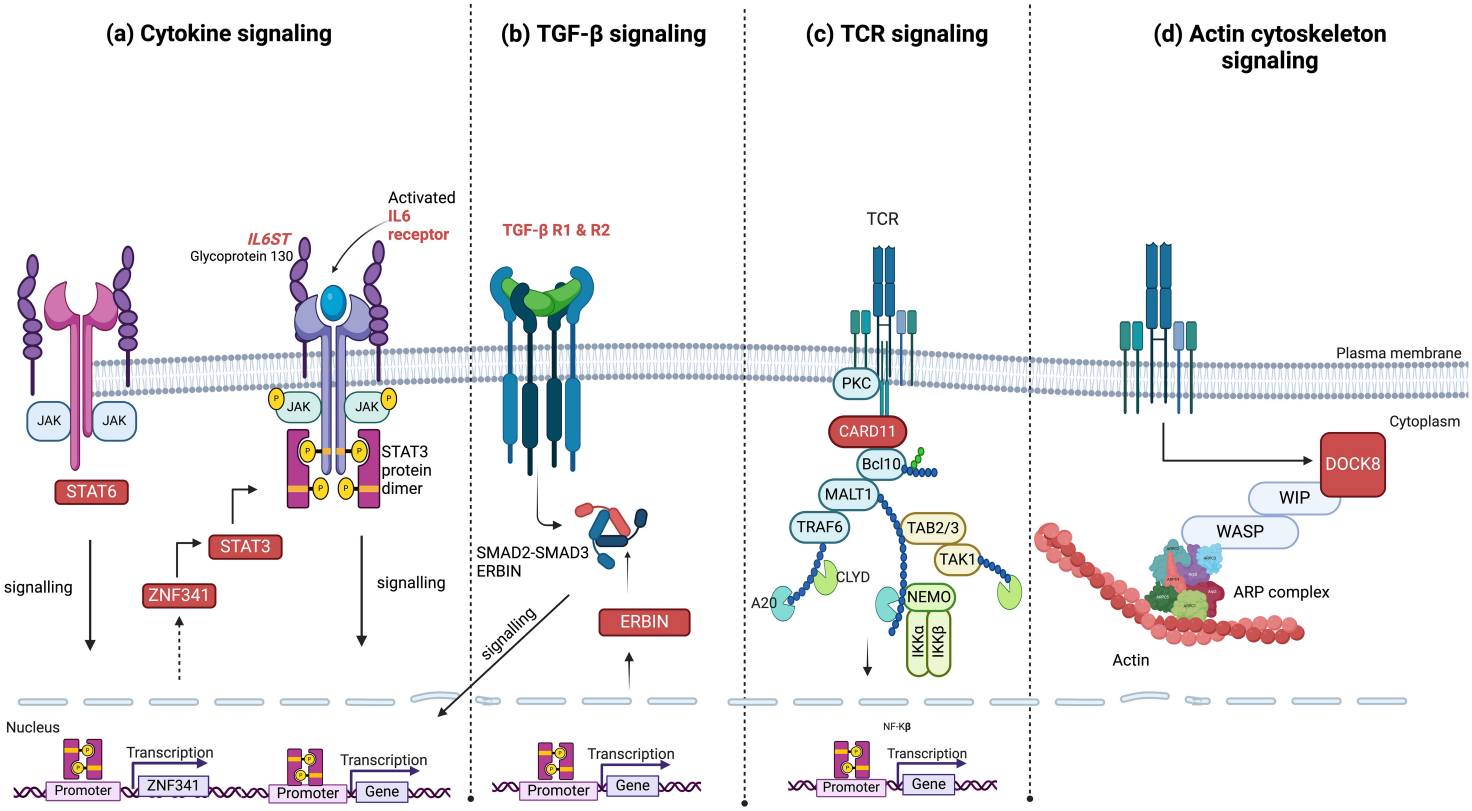
Signaling pathways and associated genes with mutations linked to hyper-IgE syndrome. **(A)** Components of cytokine signaling such as STAT3, ZNF341, STAT6, IL6R, and IL6ST; **(B)** TGF-β signaling pathway genes, including TGFBR1, TGFBR2, and ERBIN; **(C)** the antigen receptor signaling molecule CARD11; and **(D)** the actin cytoskeleton signaling molecule DOCK8. These pathways highlight the major molecular mechanisms currently identified which contribute to the clinical phenotypes of hyper-IgE syndrome. HIES genes are highlighted in red boxes or in red font and phosphorylation is indicated by yellow circles. JAK, Janus Kinase; STAT, Signal Transducer and Activator of Transcription; ZNF, zinc-finger; IL, Interleukin; ST, Signal Transducer; TGFBR, Transforming Growh Factor Beta Receptor; NF-κB, Nuclear Factor kappa-light-chain-enhancer of activated B cells; SMAD, Suppressor of Mothers Against Decapentaplegic; ERBIN, Erbb2 Interacting Protein; A20, Tumor Necrosis Factor; Alpha-Induced Protein 3; TCR, T Cell Receptor; PKC, Protien Kinase C; CARD11, Caspase recruitment domain-containing protein 11; Bcl10, B-cell lymphoma/leukemia 11; MALT1, Mucosa-associated lymphoid tissue lymphoma translocation protein 1; TRAF6, TNF receptor associated factor 6; TAB2/3, TGFb activated kinase 1 binding protein 2; TAK1, TGFb-activated kinase 1; CYLD, Cylindromatosis deubiquitinase; IKK, Inhibitor of nuclear factor kappa-B kinase; DOCK8, Dedicator of cytokinesis protein 8; WIP, WAS/WASL-interacting protein; WASP, Wiskott–Aldrich syndrome protein; ARP, actin-related protein.

In 2007, the first genetic cause of HIES was revealed as a monoallelic dominant negative (DN) missense mutation of *STAT3*, which was subsequently shown to account for over 90% of both sporadic and familial HIES cases (8). The most recent classification by the International Union of Immunological Societies (IUIS) describes eleven genetic defects associated with HIES, resulting from mutations in ten different genes. In addition to the AD loss-of-function (LOF) STAT3 deficiency, HIES encompasses a range of conditions including AR LOF IL6ST partial deficiency, AD IL6ST partial deficiency, AR IL6ST complete deficiency, AR PGM3 deficiency, AD ERBIN deficiency, AR ZNF341 deficiency, AR IL6R deficiency, AR SPINK5 deficiency, AD TGFBR deficiency (TGFBR1 and TGFBR2) deficiency, and AD CARD11 deficiency (9). Recent findings suggest that gain-of-function (GOF) mutations in *STAT6* can also cause classic hyper-IgE phenotypes (10, 11). **Figure 1** illustrates the genetic defects associated with abnormalities in T-cells in HIES. In addition to the HIES conditions reviewed here, several other PIDs associated with the onset of atopy and allergic symptoms have been identified, which fall outside of the current IUIS or Online Mendelian Inheritance in Man (OMIM) definitions for HIES (1) and detailed reviews of these conditions can be found here (12, 13).

## Autosomal dominant HIES

### AD-LOF STAT3

In 2007, it was discovered that DN mutations in Signal transducer and activator of transcription 3 (*STAT3)* were present in the majority of AD-HIES cases, thus establishing a mechanistic explanation for both the infectious and connective tissue abnormalities observed (8, 14). By 2023, around 384 patients with mutations in the STAT3 gene (OMIM: #147060) (15) had been described in the literature (16). Most of the skeletal abnormalities in patients with STAT3 deficiency, such as scoliosis and osteoporosis, are due to poor responses to leukemia inhibitory factor (LIF) (17). Asano and colleagues evaluated more than 100 known STAT3 variants, and found that 95% of the in-frame variants (128 out of 135) demonstrated dominant negative (DN) effects (18). In the context of the immune system, *STAT3* mutations impair the capacity of human CD8+ T cells to develop into granzyme B-expressing effector cells in response to IL-21 (19). STAT3 is selectively activated by certain gamma-chain (γc) family cytokines (20), particularly IL-21 (6, 21–23), whereas others mainly trigger STAT5 activation with limited involvement of STAT3 (24, 25). STAT3 is also crucial for the development of Th17 cells, a specific subset of T helper cells engaged in host defense against intracellular bacteria and anti-fungal responses. The ligands IL-6, IL-10, IL-11, IL-21, IL-22, and IL-23 transduce their signals through STAT3 (26) and as such AD LOF STAT3-HIES patients exhibit impaired cytokine signaling and defective differentiation of Th17 cells. STAT3 deficiency results in a Th1/Th2 imbalance, with a dominance of Th2 activity (27). While STAT3 is critical for IL-10 and IL-6 signaling, which regulate anti-inflammatory and pro-inflammatory pathways, respectively, STAT3-DN HIES patients exhibit defective responses to IFNγ signaling (14, 28), resulting in compromised Th1-driven immunity and a predisposition to chronic infections and inflammation. Critically, Th17 lymphocytes mediate effector responses via the release of antimicrobial peptides and production of IL-17 and IL-22 in response to bacteria, particularly *S aureus*, as well as fungi such as *Aspergillus* and *Candida.* Therefore, a deficiency in Th17 differentiation accounts for much of the increased frequency of infections in these patients (29, 30). *STAT3* LOF mutations have been observed to be closely associated with a reduced proportion of Th17 cells (less than 0.3% of CD4+ T cells), in contrast to atopic dermatitis patients with unaffected *STAT3* who generally exhibit significantly elevated levels of Th17 cells (31). Furthermore, IL-17 signaling plays a role in the regulation of neutrophil proliferation and chemotaxis. Consequently, the impaired neutrophil activity and their reduced recruitment to the lungs and skin could explain the recurrent staphylococcal infections observed in these areas (32–34). Additionally, STAT3 is crucial for the development, differentiation, and maintenance of memory T cell, and individuals with HIES show a reduced number of CD4 and CD8 central memory T cells (35, 36). STAT3-deficient patients have been shown to exhibit a diminished capacity of CD8+T cells to manage herpesvirus infections (37), which may be explained by the observation that in STAT3 deficient patients, there are increased levels of naïve CD8 T cells, and a concomitant decrease in central memory, effector memory, and terminally differentiated effector memory cytotoxic CD8 T cells (19).

Additionally, STAT3 plays a key role in naïve B cell activation, affinity maturation and class switching during the induction of humoral immune response (38, 39). In B cells, STAT3 signaling governs the structure and recycling of maturing cells through the germinal center light and dark zones, as well as the exit of mature cells from germinal centers, providing a mechanistic explanation of how STAT3 defects adversely affect production of memory B cells (40), leading to a reduction in the maintenance of circulating memory B cells. Although, *STAT3* LOF mutations lead to a dysregulation of B cell responses, pateints typically have normal levels of total serum IgM, IgG, and IgA, whilst exhibiting hyper-elevated levels of serum IgE, impaired antigen-specific antibody responses, and decreased memory B cell levels (3, 41–45). Considering the importance of STAT3 for both plasma cell differentiation (IL-21 signaling) and CD4+ T-follicular helper cell (Tfh) (IL-6 signaling), IgA, IgG and IgM levels are found to be surprisingly within the normal range in many cases (46). However, there is a notable reduction in vaccine responses, linked to the noted deficiencies in B-cell maturation and as seen by a decrease in CD27+ switched memory B cells (44). Immunoglobulin replacement therapy (IgRt) has shown a positive impact on the improvement of *S. aureus* infections in individuals with STAT3-HIES (3, 31, 44). Studies indicate that IgRT can enhance the levels of *S. aureus*–specific IgG levels in STAT3-HIES patients (47). Studies reported the association of STAT3 mutations and impaired memory B cell function (48, 49). The National Institutes of Health (NIH) scoring system, assesses the presence and severity of 21 clinical and laboratory measures and was created for the purpose of identifying AD-HIES (50). 96% of the 48 AD-HIES patients with *STAT3* mutations present in an observational study achieved a score of ≥40, which indicates the diagnostic utillity of this scoring system (31). In young patients, the score may be lower due to the absence of certain clinical features (51), such as scoliosis, fractures, and facial dysmorphic traits, which typically appear and become more pronounced as they grow and the full phenotype emerges over time.

Clinically having a normal IgE level or one below 2000 kU/I is not necessarily exclusive of the diagnosis (46). Eosinophilia is found in over 90% of STAT3-DN patients and is linked to impaired neutrophil function (52), and the induction of neutropenia with circulating neutrophils decreasing to approximately 700 cells per microliter. Interestingly, IgE levels and eosinophilia do not neccesarily predict the severity of the disease (50, 53).

Syndromic connective tissue features linked to *STAT3*-HIES exhibit variability: the characteristic facies, include a high-arched mouth, expanded nasal bridge, deep-set eyes, and large forehead, which typically manifest during adolescence, may not appear in early childhood (54). Most children need to have their primary teeth extracted, due to a lack of root resorption in the primary teeth, which is required for eruption (55). One-third of patients have joint hyperextensibility and degenerative joint disease manifesting in adulthood. Moreover, there is a predisposition to early-onset long bone fractures with minimal trauma. In 79% of cases, there is a decrease in bone mineral density (BMD), which does not correlate with the rate of fractures. While lower BMD is shown in the femoral neck and spine in classical osteoporosis (56), in *STAT3*-HIES, only radial BMD is associated with the risk of fractures (57). Vasculopathy in STAT3-HIES presents significant challenges for both understanding its pathogenesis and managing it clinically (58). Predominantly, medium-sized arterial abnormalities are observed, particularly affecting the coronary and intracranial vessels. In a prospective study, 50% of patients showed coronary artery abnormalities with ectasia and aneurysms being the most prevalent findings, alongside radiographic evidence of small infarcts in some cases (53). Mutations in the *STAT3* gene have additionally been linked to various malignancies (16). Patients with HIES face a markedly elevated risk of developing aggressive B cell lymphomas, potentially due to defects in the STAT3/IL-21 pathway influencing B cell differentiation into plasma cells, with potential involvement of T follicular helper cells (45, 59). A study of 65 AD-HIES patients (including 35 with DNA-binding mutations and 30 with SH2 mutations) examined clinical characteristics using the NIH HIES scoring system alongside findings such as brain and coronary artery abnormalities. While patients with SH2 mutations exhibited a higher occurrence of certain features, such as a high palate, wider inter-alar distance, upper respiratory tract infections, and scoliosis (particularly in pediatric cases), individuals with DNA-binding mutations showed a higher risk of mortality (60). Recent cases, including an 18-year follow-up and seven additional patients, have shown improved survival and significant alleviation of disease-related complications (61). Allogeneic hematopoietic stem cell transplantation (HSCT) is an option to treat STAT3 deficiency, especially the immunological elements of the condition and stabilize even severe lung complications and abscesses and recurring skin infections. Notably, Th17 cells from donor cells may generate comparable amounts of IL-17A to those of healthy controls. However, long-term monitoring is necessary for determining the extent of syndromic reversal due to the broader functions controlled by STAT3 (61, 62).

### IL6ST-DN

*IL6ST* encodes Glycoprotein 130 (GP130), the common subunit of the signaling receptors for all ten IL-6 family cytokines (2, 63). Stimulation of these cell-membrane receptors triggers JAK/STAT pathway, essential for transmitting signals to the nucleus across multiple cell types and plays a major role in the regulation of T helper differentiation (64). A study involving 12 patients from 8 unrelated kindreds with AD-HIES caused by DN IL6ST mutations (OMIM: #619752) revealed that natural killer (NK) cell counts, and differentiation were normal in all patients, except for a few who exhibited low NK cell counts. CD4+ and CD8+ T cell counts were generally normal, however, one patient had low CD8+ T cell counts, while another had high CD4+ T cell counts. In the CD4+ and CD8+ T cell compartments, patients showed high naive T cell frequencies, but there were low frequencies of central memory T cells observed in the CD8+ compartment. Additionally, the CD8+ T cells contained a low proportion of effector memory T cells. The proportions of γδ T cells, invariant natural killer T (iNKT) cells, and regulatory T (T reg) cells remained normal, whereas the proportion of mucosal-associated invariant T (MAIT) cells was low. Within the memory CD4+ T cell compartment, the frequency of T follicular helper (Tfh) cells was low, whereas T helper type 1 (Th1), and Th17 cell frequencies were normal, with very high frequencies of Th2 cells (65).

During adaptive immune response, IL-6 plays a significant role in stimulating both antibody production and the development of effector T-cells (66). Pro-inflammatory cascades and the activation and differentiation of both B and T cells are disrupted in the absence of functional IL-6 signaling (67). IL-10, synthesized by regulatory T cells and B cells, has been linked to changes in production of IgG4 rather than IgE (68, 69), whereas IL-21 regulates both the induction and inhibition of IgE production (70, 71). Individuals with IL6ST deficiency often exhibit elevated levels of IgE due to the involvement of IL-6, IL-11, and IL-27 and their influence on other cytokine signaling pathways such as IL-10 and IL-21 (67, 72). IL-6 is believed to regulate IgE production through acting directly on B cells and indirectly on CD4+ T cells (73). Patients with autosomal recessive IL-10 or IL-10R deficiency maintain IgE levels within normal reference ranges (74, 75), suggesting that defective IL-10/STAT3 signaling does not impact class switch recombination (CSR) to IgE (73). Individuals with IL-11RA deficiency present with craniosynostosis and dental anomalies, but do not exhibit significant immunodeficiency (76). Therefore, IL-6 is considered the principal cytokine activating STAT3 that inhibits IgE production in human B cells (73). The hyper-IgE phenotype and clinical features of atopic disease, like dermatitis, food allergy, and eosinophilia, seen in STAT3 deficiency and ZNF341 deficiency are replicated by pathogenic variants in IL6R or IL6ST (17, 67, 77–80).

In mouse models, a complete deficiency of gp130 is lethal at the embryonic stage, while complete *IL6ST* LOF in humans results in the lethal Stüve-Wiedemann syndrome, defined by neonatal lung dysfunction, defective acute-phase response, skeletal dysplasia, renal abnormalities, atopic dermatitis, and congenital thrombocytopenia (81). Heterozygous DN *IL6ST* mutations were identified in 12 patients belonging to eight unrelated families, who exhibited clinical characteristics strikingly similar to *STAT3*-DN HIES, involving pneumatocele. Individuals with *STAT3* and *IL6ST* mutations exhibit infectious and allergic symptoms characteristic of IL-6R deficiency, as well as some of the skeletal deformities present in IL-11R insufficiency. Thus, DN *STAT3* and *IL6ST* mutations result in similar clinical phenocopies due to the disruption of the IL-6 and IL-11 response pathways (17). Patients who lack IL6ST suffer from impaired innate immune response to bacterial infections, which can cause acute-phase reactions to be delayed or completely absent (82). IL6ST-DN HIES patients also exhibit non-immunological characteristics such as scoliosis, osteoporosis, and retention of primary teeth. In contrast to individuals with partial LOF *IL6ST* mutations, complete IL11RA deficiency, or *STAT3*-DN mutations, individuals with IL6ST-DN do not exhibit craniosynostosis, most likely because of remaining functionality in IL-11 signaling (76). The skeletal phenotype is probably influenced by impaired IL-11 signaling, given that individuals with *IL11RA* mutations manifest with craniosynostosis and dental malformations (76, 83). Patients with DN mutations in IL6ST underwent treatment with subcutaneous immunoglobulin treatment (17). The analysis of *IL6ST* mutations brought to light that IL6ST/IL6R cytokine receptor complex formation is essential for the signal transduction upstream of STAT3 in HIES pathogenesis. After JAKs are phosphorylated by the hexameric IL6-IL6R-IL6ST complex, tyrosine residues within the cytoplasmic domain of GP130 are phosphorylated by JAKs (**Figure 1**), which leads to the attraction and activation of STAT1, STAT3, and STAT5. Subsequently, the phosphorylated STATs form dimers and migrate into the nucleus to interact with the promoters of acute-phase protein genes that contain IL-6-responsive regions. Hence, mutations in the IL6R, IL6ST, and STAT3 genes all lead to disruption of IL-6 family signaling pathways, but with distinct differences in their clinical manifestations (1).

### AD TGFBR deficiency (TGFBR1 and TGFBR2)

*TGFBR1* and *TGFBR2, GOF* mutations (OMIM: #609192) and (OMIM: #610168) (84), were the first documented genetic causes of Loeys–Dietz syndrome (LDS) (85–88). LDS was first described in 2005 as an autosomal-dominant connective tissue disorder associated with aortic aneurysms, hypertelorism, widespread arterial tortuosity, and bifid/broad uvula or cleft palate (84, 89). As of now, 53 patients with Loeys-Dietz syndrome have been identified in published studies (90–92).

TGF-β signaling can influence the activation and differentiation of T cells, and the process of immunoglobulin class switching by B cells following allergen exposure (93–95). The ability of regulatory CD4+ T cells (Treg) to produce TGF-β plays a significant role in their ability to maintain homeostasis and prevent allergic disease and autoimmune responses (96).

Dysregulated TGF-β signaling in LDS patients increases serum IgE concentration through dysregulated CD4+ T cell differentiation and elevation in Th2 cytokine levels, leading to the induction of allergic reactions similar to those seen in STAT3-HIES (1, 2). LDS mutations promote TH2 skewing in naïve lymphocytes through a cell-autonomous mechanism. Naïve CD4(+) T cells from LDS patients, unlike those from controls, exhibited an accumulation of TH2 cytokine-producing cells following stimulation with TGF-β (97). In agreement with the role of TGF-β in promoting the generation of Treg cells (98), but inconsistent with the observed loss of tolerance, LDS patients exhibited an increased frequency of peripheral FOXP3+ Treg cells with intact ability to suppress effector T cell proliferation (97). Additionally, TGF-β can exert its effects on mast cells during an acute allergy episode to regulate the severity of the reaction. This is particularly relevant in hyper-IgE syndromes, which are marked by dysregulated mast cell function and intensified allergic responses. TGF-β also plays a role in promoting tissue remodeling following the damage resulting from an allergic attack (95). TGF-β signaling serves as a crucial connection between the function of vascular smooth muscle cells (SMCs) and the composition of the extracellular matrix (ECM). LOF mutations resulting in TGF-β signaling pathway genes have been linked to alterations in aortic SMC phenotypes, causing structural alterations in the aorta and contributing to hereditary connective tissue disorders (99). Variants in TGF-β pathway have been associated with atopic conditions. Haque et al. investigated the role of TGF-β signaling in IgE production in allergic diseases by studying both patients with Loeys-Dietz Syndrome (LDS) and mice with specific gene defects causing partially reduced canonical TGF-β signaling. CD4+ T cells from both LDS patients and affected mice demonstrated impaired TGF-β signaling, which was correlated with atopy. The mutations seen in LDS led to an increase in T follicular helper 2 cells, heightened humoral immune responses, and the production of allergen-specific IgE. Additionally, LDS variants caused dysregulation PI3Kγ/mTOR signaling, which was able to be corrected through pharmacological inhibition (100). Immunological tissues from HIES individuals with TGFBR1/2 mutations display an increased accumulation of phosphorylated suppressor of mothers against decapentaplegic homolog 2 (SMAD2) in the thymus (1). This disrupted signaling affects downstream pathways, which helps to explain the increased allergic reactions and connective tissue issues observed in these patients.

LDS is characterized by rapidly progressing aortic aneurysmal disease, making close surveillance crucial (84, 89). Regular echocardiography is essential to evaluate he aortic root, ascending aorta, and heart valves in affected individuals (101). In LDS patients, sinusitis and otitis may occur together with mucus buildup due to the heightened allergic reactivity and malformed craniofacial structures (2). An instance of febrile status epilepticus linked to COVID-19 in an infant with Loeys-Dietz syndrome (LDS) has been reported (102). A case has also been reported of a patient with LDS who developed annular aortic valve abscess and ascending aortic dissection caused by *Staphylococcus aureus* endocarditis (103). Additionally, LDS is associated with several atopic conditions including eosinophilic gastrointestinal disease, allergic rhinitis, atopic dermatitis, food allergy, and asthma (1, 2).

### AD ERBIN deficiency

*ERBIN2IP* encodes ERBIN (OMIM: *606944), which plays a crucial role in regulating TGF-β associated activities. ERBIN is essential in facilitating the crosstalk between STAT3 and TGF-β signaling. ERBIN is activated via STAT3 whereupon they form a complex that inhibits SMAD2/3 nuclear localization and TGFBR signal propagation (104).

Unlike *TGFBR* deficiency, which alters TGF-β receptor signaling directly, STAT3 activation of ERBIN in individuals with *STAT3* or *ERBIN2IP* LOF mutations may induce an increased level of TGF-β activity. This results in increased IgE and Th2 cytokine expression as well as excessive activation of IL-4/IL4R (2). ERBIN defective memory CD45RO+ CD4+ cells produce high levels of Th2 associated cytokines (IL-4, IL-5, and IL-13) *ex vivo*, which accounts for the allergic symptoms (105). Under homeostatic conditions, TGF-β signaling is essential in preventing the spontaneous development of allergic disorders such as asthma, eosinophilic esophagitis, and atopy in mouse models (106). Another important result of this heightened signaling is the elevated expression of IL-4 and IL4Rα (107).

So far, only 3 cases of ERBIN deficiency have been reported (108, 109). Patients with *ERBIN* or *STAT3* mutations commonly exhibit severe eosinophilic gastrointestinal illness (EGID), bacterial infections, allergen-specific reactivity, hyper-IgE, and connective tissue anomalies, including aneurysms and joint hypermobility (108). Scoliosis, a significant connective tissue anomaly observed in individuals with LDS and those with *STAT3*-DN mutations, is frequently associated with genetic variations in the *ERBIN* gene (110). Despite similarities in connective tissue symptoms between *STAT3* DN, *TGFBR* LOF, and *ERBIN* LOF mutations, further insights into the syndrome specific effects may be provided by a deeper analysis in the differences induced in the TGF-β signaling pathway (107). Further, *ERBIN* mutations could also play a role in specific epithelial disease types like atopic diathesis (104).

Notably, administration of IL-4Rα blockade (using biologic therapeutics like dupilumab) to patients with *STAT3* DN and *ERBIN* LOF conditions shows considerable improvement in otherwise unresponsive cutaneous and gastrointestinal inflammation in these patients (105, 111–116). Targeting type 2 signaling through dupilumab treatment in a patient with *ERBIN* deficiency effectively controlled co-existing allergic inflammation, suggesting a possible treatment strategy for comorbid atopy in other individuals with disrupted TGF-β signaling pathways (105).

### CARD11-associated atopy with dominant interference of NF-κB signaling

Heterozygous variations can either enhance or significantly impair CARD11 functional efficiency, given that CARD11 oligomerization is necessary for downstream signaling (117). Germline *CARD11* mutations lead to at least three differentially characterised PIDs, including severe atopic disease (dominant interfering, heterozygous and loss-of-function mutations), B cell expansion with NF-κB and T cell anergy (BENTA; heterozygous, gain-of-function mutations), and severe combined immunodeficiency (biallelic null mutations) (118–120). In 2017, it was shown that a form of AD-HIES with atopy and hypogammaglobulinemia is driven by heterozygous LOF DN mutations in the *CARD11* gene, also referred to as immunodeficiency 11B with atopic dermatitis, (OMIM: #617638) (121). CARD11-associated atopy with dominant interference of NF-κB signaling (CADINS), was first identified in 2017, and is clinically defined by the presence of atopy and a variety of autoimmune and/or infectious symptoms (122). Also, it has been reported that heterozygous LOF *CARD11* mutations, which function as strong DN alleles, are the cause of this condition (123).

To date, 62 patients have been identified with 17 distinct dominant-negative mutations in the *CARD11* gene, with most mutations located in the N-terminal CARD and CC domains (118, 121, 122, 124–128). CADINS symptoms, can include asthma and other allergic diseases, autoimmune diseases, and infections including respiratory tract and viral skin infections. Significant variations in the incidence of symptoms have been noted between kindreds and even within family members (126). Due to the accompanying B and/or T cell defects, CADINS patients may experience a more severe infectious phenotype than those with classical atopic dermatitis (125). Clinical symptoms observed have been linked to specific immunologic defects in patients including neutropenia, hypogammaglobulinemia, aberrant T-cell proliferation and differentiation (122). Further, CARD11 mutations may additionally predispose patients to an increase incidence of malignancies (16). Although IgE levels are high in all CADINS patients, these are generally lower than those seen in STAT3 deficiency (46, 118). The presence of eosinophilia is a recurrent finding (46) and a study involving 15 CADINS patients revealed that the majority exhibited consistent eosinophilia (80%) (125). Quantitative immunoglobulin levels for IgM, IgG and IgA are generally within normal ranges, with only sporadic observations of slightly reduced IgG levels and elevated IgA levels. Vaccine-induced antibody response may exhibit variability, notably response to carbohydrate antigens are consistently suppressed (46). Patient B- and T-cell counts are within the normal range, while phytohemagglutinin (PHA) and anti-CD3-induced cell proliferation has been shown to be reduced. Whilst, AD-CARD11 individuals share a lot of similarities with DOCK8-deficient patients in terms of clinical and immunological characteristics, mucocutaneous Candidiasis and cutaneous viral infections occur less frequently, and only one affected patient has presented with neurologic sequelae (seizures and nystagmus) (46). According to reports, individuals with CARD11 deficiency may experience improvements when treated with intravenous immunoglobulin (IVIG) or Subcutaneous immunoglobulin (SCIG) because it would lower their infection rates (46).

### AD STAT6 GOF

STAT6 is critical for propagation of signaling from the IL-4 and IL-13 receptors to the nucleus in order to drive a multitude of effects, chiefly directing Th2 differentiation in naïve cells and inducing IgE class switching in B cells (129). As such, there is a close connection between STAT6 and the biology that induces allergic inflammation. The primary and most well researched function of STAT6 involves facilitating the biological impacts of IL-4, an essential cytokine required for various processes such as M2 macrophage polarization (10, 130), Th2 cell differentiation, as well as B cell survival, proliferation, and class switching to produce IgE (131, 132). STAT6 activation in T cells leads to the induction of GATA3 expression, the master regulator of Th2 differentiation. Increased Th2 differentiation subsequently lead to increases in production of IL-4, IL-5, and IL-13, cytokines essential for inducing allergic reactions by stimulating downstream innate immune responses in the form of mast cells, eosinophils, basophils and innate lymphoid type-2 cells (ILC2) (133, 134). For both acute and chronic allergic disease symptoms, elevated IgE in conjunction with increased levels of mast cells and basophils which express high affinity immunoglobulin E receptor (FcER) is a critical component, which may additionally contribute to the allergic predisposition and the atopic march (135). Elevated TH2 cell populations or TH2 cells that secrete large amounts of IL-4, IL-5, and IL-13 may underlie the allergic phenotype observed in the STAT6 GOF patients (10). Overactive STAT6 signaling in airway epithelial cells and dendritic cells can foster conditions that support asthma and chronic lung diseases by driving the production of chemokines that attract TH2 cells and eosinophils (6, 136, 137). This highlights how dysregulated STAT6 activity contributes to the development of allergic and chronic inflammatory diseases, underscoring its potential as a therapeutic target for managing these symptoms.

Recently, 21 individuals with severe allergic disease from birth were diagnosed with STAT6-GOF disease (OMIM: #620532) (130). While the complete spectrum of characteristics exhibited by individuals with GOF STAT6 variants remains to be fully elucidated pending identification of more affected individuals, current clinical indicators include early onset of elevated levels of serum IgE, increased eosinophils in peripheral blood. Patients may also present with persistent and widespread atopic dermatitis resistant to treatment, recurring infections affecting the skin and respiratory system, multiple allergies to both food and drugs. Severe cases of anaphylaxis (sometimes fatal), eosinophilic gastrointestinal disorders, allergic rhinoconjunctivitis, asthma are frequently reported. Other notable features may include shorter stature and potentially cerebral vascular anomalies, although further research is needed to confirm these findings (10, 11). A case has been reported of a family with STAT6 GOF disease presenting with severe early-onset atopy and follicular lymphoma and Linked to a newly identified germline heterozygous STAT6 mutation, c.1255G>C p.D419H, residing in the DNA binding domain of STAT6 and co-segregating with the clinical phenotype (138). Dupilumab and tofacitinib treatments have demonstrated efficacy in the treatment of STAT6 GOF patients (10).

## Autosomal recessive HIES

### DOCK8 deficiency

In 2009, mutations in *DOCK8* (OMIM: #243700) were identified as the predominant cause of AR-HIES (139, 140). Based on the update of IUIS phenotypical classification in 2022, AR DOCK8 deficiency was reclassified as a B cell low subgroup of combined immunodeficiency (CID) (141). DOCK8 belongs to a protein family containing 11 members having major roles in control of cell adhesion, migration, and morphology. DOCK8 has a central role in regulating the reorganization of the actin cytoskeleton during migration and synapse formation, and immune abnormalities associated with DOCK8 deficiency are linked to these functions (2). Around 230 patients of DOCK8 deficiency have been reported by 2017 (142).

Studies assessing immune function in mice, lacking DOCK8, found abnormalities in dendritic cell (DC) functionality, showing DCs were unable to accumulate in draining lymph nodes, thereby reducing T-cell priming (143). Additionally, DOCK8 serves as an intermediary protein in B cells, functioning upstream of STAT3 and downstream of toll-like receptor 9 (TLR9), facilitating the proliferation of B cells and the generation of immunoglobulins (144). Studies of both humans and mice with DOCK8 inactivating mutations have demonstrated that DOCK8 is involved in the survival of naive CD8 T cells, the polarization of lymphocyte function–associated antigen 1 (LFA-1) towards the immune synapse, and the memory and recall responses of CD8 T cells following viral infection (145). Lack of DOCK8 may affect long-term B cell memory (145, 146). DOCK8 mutant B cells were unable to develop into marginal zone B cells or sustain their presence in germinal centers during affinity maturation. DOCK8 mutation disrupted the concentration of Intercellular Adhesion Molecule 1 (ICAM-1) in the B cell immune synapse, but did not change other features of B cell antigen receptor signaling. The development of a humoral immunodeficiency caused by a DOCK8 mutation suggests that the arrangement of an immune synapse is essential for signaling and the survival of those B cell subsets needed for sustaining long-lasting immunity (147). As CD8+ T-cells have essential activity in antiviral defense, in case of DOCK8 deficiency, CD8+ T-cells exhibit significant decreases in IFN-γ and TNF-α production as well as CD8+ T cell proliferation (2). DOCK8 deficiency may affect survival of memory CD8+ T cells specific to viruses, which potentially explains the increased susceptibility to bacterial and chronic viral infections (145, 146). Furthermore, leukocyte migration to infected skin is impaired (2). LFA-1 is a key adhesion molecule that plays an essential role in T cell migration and retention within lymph nodes (148, 149). Moreover, the interplay between LFA-1 and ICAM-1/2 on B cells is essential for the positive Tfh–B cell contacts that result in B cell clonal growth (150). DOCK8 expression in T cells is crucial for LFA1-dependent positioning within germinal centers (GCs), GC B cell production, and IgG antibody responses to T cell-dependent antigens (151). Therefore, the effectiveness of adaptive immunity is significantly diminished in individuals with DOCK8 deficiency because the accumulation of adhesion molecules and cytotoxic granules at immunologic sites is hindered (2). In DOCK8 deficient individuals, T cells exhibit a skewing towards Th2 differentiation, leading to subsequent increases in IgE levels (152). *In vivo* studies have revealed cell death as a crucial signal leading to the Th2-skewed CD4+ T cell response in DOCK8-deficient mice. Here, lung-infiltrating DOCK8^−/−^ mononuclear phagocytes expressing the fractalkine receptor CX3CR1 exhibit heightened susceptibility to migration-induced shattering (cytothripsis), which releases IL-1β and causes CD4+ T cells to produce granulocyte-macrophage colony-stimulating factor (GM-CSF). Caspase-dependent cell death, in conjunction with IL-1β, is also a crucial driver for the Th2 bias (152).

In addition to the systemic manifestations shared by many combined immunodeficiencies, a key characteristic of DOCK8 deficiency is the elevated incidence of skin infections and eczema (153). From a clinical perspective, patients with DOCK8 deficiency have symptoms resembling those of AD-HIES, such as recurrent sinopulmonary infections, mucocutaneous *Candidiasis*, *Staphylococcal* skin abscesses, heightened IgE levels, eczema, eosinophilia, and an increased likelihood of developing malignancies (154). Compared to other PIDs (even those with significantly elevated IgE levels, such as DN mutations in *STAT3*), DOCK8 deficiency leads to an extremely high frequency of severe food allergies which is a distinctive aspect of DOCK8 deficiency (140, 144, 155–157). DOCK8 deficient individuals have in contrast been found to exhibit far lower rates of skeletal and connective tissue anomalies, specifically the characteristic facies, retained dentition, and minimal trauma fractures commonly observed in AD-HIES (143). Patients with DOCK8 deficiency are also vulnerable to viral infections such as *human papilloma virus* (2), which are thought to play a significant role in the increased frequency at which they develop malignancies, with squamous cell carcinoma and lymphoma being the primary types, affecting around 17% of patients (158). Other types of cancers, including micro-cystic adnexal carcinomas and cutaneous T cell carcinomas, are unrelated to viral infections (159). While *STAT3*-HIES patients frequently live into their sixth decade, the median lifespan in *DOCK8* patients, despite the use of preventive anti-microbial measures and immunoglobulin replacement therapy, is typically 20 years (4). Studies have shown that HSCT is effective as a cure for most patients with DOCK8 deficiency, establishing this method as the treatment of choice (160, 161). Also, dupilumab was effectively employed to achieve remission of chronic widespread eczema herpeticum in a patient with hyper-IgE syndrome due to DOCK8 deficiency (162).

Although NIH scores exceeding 40 are sometimes obtained, such occurrences are less common compared to individuals with AD-STAT3 deficiency (46). While both conditions exhibit increased serum IgE levels and eosinophilia, primarily because of decreases in T cells, patients with DOCK8 deficiency display decreased serum IgM levels and lymphopenia (154). Normally, IgG and IgA levels are within the normal range or increased (163), and a reduction in B-cells is less common (46), poor vaccination reactions are a commonly observed problem (155, 163).

### ZNF341 deficiency

Two studies published in 2018 described biallelic LOF variants of zinc finger protein 341 (*ZNF341)* (OMIM: #618282), which encodes a protein with a previously unknown function and linking it to a new AR-HIES. The truncated forms of ZNF341 were found to be responsible for inducing a STAT3-like hyper-IgE phenotype (164). These studies derived the role for ZNF341 as a transcription factor responsible for regulating both the inducible and baseline levels of *STAT3* expression (164, 165).

Compared to those with *STAT3*-DN HIES, *ZNF341* patients exhibited fewer non-immunological symptoms and more robust inflammatory reactions (1). Here, IL-6 family member receptors such as the IL-11 receptor, which contributes to the joint, skeletal, tooth, and vascular symptoms of *STAT3*-HIES, may not exert the same level of influence as they present in STAT3 deficiencies, suggesting that ZNF341 has additional activities which are independent of the STAT3 signaling pathway (2). Similar to *STAT3* LOF mutations in HIES patients, the disruption of ZNF341 signaling leads to an inhibition of Th17 differentiation and results in less IL17 and IL-23 production by CD4+ memory and effector T cells, due to defects in STAT3 dependent signaling (164).

Whilst individuals affected by AR-ZNF341 deficiency exhibit several clinical and immunological features similar to those with AD-STAT3, their clinical symptoms tend to be milder. However, NK cell numbers are notably lower in this patient population (166). Currently, there is a lack of correlation between ZNF341 deficiency and common atopic symptoms such as asthma, dermatitis, rhinitis, and food allergies. A potential explanation for this observation is that ZNF341 with its 12 zinc fingers functions as the activator of cytokine-mediated STAT3 production. This suggests that zinc finger transcription factors can recruit transcriptional activators and repressors without functional domains (2). An adult patient with ZNF341 deficiency and severe atopic dermatitis showed significant clinical improvement and a reduction in IgE levels after treatment with dupilumab (167). ZNF341 also serves as an enhancer of *STAT3* expression, and homozygous nonsense mutations in *ZNF341* result in inadequate *STAT3* expression. This insufficiency is likely a contributing factor to the symptoms of HIES. However, further research is needed to understand the precise role of ZNF341 in the pathogenesis of *ZNF341* LOF HIES, as there is not conclusive evidence showing that reintroducing normal *ZNF341* restores STAT3 expression and signaling. Additionally, the STAT3-independent functions of the ZNF341 may also play a role in the syndromes’ manifestation (1). To date, 20 patients with ZNF341 deficiency have been reported (166). The overall number of reported patients is very limited, which constrains the understanding of both the clinical features and immunophenotype associated with this condition.

### PGM3 deficiency

Hypomorphic autosomal-recessive mutations in *PGM3* (OMIM: #615816) can result in a glycosylation disorder that exhibits a variety of clinical manifestations with varied penetrance and expressivity. Additional reported issues include, connective tissue abnormalities, immune dysregulation, and impairments in neurodevelopment (104). Clinical manifestations in patients with PGM3 deficiency include recurrent infections (particularly *Staphylococcus aureus)*, *Candidiasis*, or viral etiologies in the skin, respiratory tract, or gastrointestinal tract, atopy, neurologic symptoms, enteropathy, bone marrow failure, skeletal dysplasia (168–170), as well as hyper-elevated serum IgE (168). Severe combined immunodeficiency (SCID), neutropenia, and skeletal anomalies can also be present in infants (170). Neurocognitive impairments and likely hypomyelination, which are not observed in cases of STAT3 or DOCK8 deficiency, are distinct features of PGM3 deficiency (171). Vascular abnormalities have been observed in PGM3-deficient patients, although they are uncommon (168). By 2014, a total of 40 cases from 18 families with PGM3 deficiency had been documented in the literature (168–170, 172–178). Due to the significant overlap with AD-HIES, a large percentage (87%) of these patients have an NIH score of ≥40, higher than generally observed in DOCK8 deficiency (168, 169).

Patients with PGM3 deficiency commonly experience neutropenia, reduced memory B-cell, and T-cells counts. Nonetheless, they also exhibit hypergammaglobulinemia, while having intact antibody responses to protein and carbohydrate antigens (168, 169). Antibody levels against carbohydrate and protein antigens are generally sufficient for protection. T-cell lymphopenia is a common finding affecting predominantly the CD4+ subset, leading to an inverted CD4:CD8 ratio (169). While the PGM3 protein levels may appear normal, analysis of activity in PGM3-deficient fibroblasts can show a distinct reduction, and serves as an additional relevant diagnostic criterion (168). Individuals with PGM3 deficiency may potentially improve the catalytic activity of PGM3 when an enzymatic route is blocked by supplements such as N-acetyl-glucosamine (GlcNAc), which leads to a reduction in pathologic phenotypes (108). Additionally, reports on the outcomes of HSCT in PGM3 deficiency, though limited, highlight that hematopoietic stem cell transplantation with cord blood or bone marrow from matched related donors led to successful engraftment and resolution of both neutropenia and lymphopenia (170). These findings underscore the potential of HSCT as a therapeutic option for this rare disorder. Along with HSCT, immunoglobulin replacement therapy and antimicrobial prophylaxis are key treatment approaches for PGM3 deficiency patients (170, 174, 179).

### IL6R-LOF

LOF mutations in *IL6R* (OMIM: #618944) were first observed in two unrelated patients, who exhibited atopic dermatitis, recurrent skin and lung infections, and reduced inflammatory responses. These mutations were found to be responsible for encoding an IL-6R ligand binding component. The patients experienced repeated skin abscesses caused by *S. aureus*, upper and lower respiratory tract infections, though intriguingly their levels of C-reactive protein (CRP) were only slightly elevated during acute infection (78). Four additionally identified patients exhibited impaired B-cell class-switching and antibody production, a tendency toward severe invasive bacterial infections, severe eczema, absence of acute-phase reactant responses, and potential elevation in IL-6 production (79). The impairment of cellular responses to IL-6 alone may account for most of the characteristics of the primary HIES (180). Immunoglobulin replacement therapy is a cornerstone treatment for patients with bacterial infections and hypogammaglobulinemia, along with antimicrobial prophylaxis (181). In severe cases involving pronounced immunodeficiency and recurrent life-threatening infections, hematopoietic stem cell transplantation (HSCT) might be considered (182). However, contrary evidence exists as patients with neutralizing autoantibodies against IL-6 have symptoms comparable to those of IL-6R LOF, specifically reduced inflammatory response but not atopic manifestations. Around 10 patients with IL-6R deficiency have been documented in the literature (183). Further research is required, especially to determine the exact molecular mechanism by which IL-6 and/or other signaling molecules control atopic features (1).

### IL6ST-partial LOF

N404Y and P498L were the first to be identified as novel homozygous missense mutations in *IL6ST* (OMIM: #618523) from two patients of South Asian and Turkish heritage respectively. To date, a partial loss-of-function mutation in the IL6ST gene have been identified across 20 patients (184). Validation of the putative defects were confirmed as partial signaling defects after evaluation of the functionality of reconstituted IL-6 receptor complexes in HEK 293 cells lacking intrinsic GP130, following transfection with patient *IL6ST* sequences (67, 80). When patient-derived fibroblasts were stimulated with IL-6 and IL-11, the response of STAT3 was significantly diminished and the deficiency was repaired when wild-type GP130 was expressed. Both patients exhibited eczema, eosinophilia, elevated serum IgE levels, scoliosis, recurring lung infections, decreased acute-phase responses, retained deciduous teeth as well as craniosynostosis (1).

Patient P498L was treated with monthly IVIG (80). These results imply that IL6ST plays a predominant role as cytokine receptor upstream in the pathophysiology of *STAT3*-DN HIES (1).

### SPINK5

Comel-Netherton syndrome is a result of AR mutations in *SPINK5* (OMIM: #256500) and is an extremely rare condition with an incidence of 1/200,000 (185). *SPINK5* produces the lymphoepithelial Kazal-type-5 related inhibitor, which plays a role in regulating the process of desquamation. Symptoms typically include an ichthyosiform erythroderma, presenting early in life, with many patients going on to acquire distinctive syndromic features such as ichthyosis linearis circumflexa and anomalies of the hair shaft (easily broken hairs or bamboo hair). As of 2021, more than 200 cases of Netherton syndrome have been reported (186–191). Comel-Netherton patients are also likely to experience atopic symptoms, including anaphylaxis, severe atopic dermatitis, hay fever, and food allergies (46).

Comel-Netherton syndrome patients were shown to have a reduced proportion of both non-switched and class-switched memory B cells, deficiencies in NK cell cytotoxicity, and to exhibit a limited response to the T-cell-dependent neoantigen, bacteriophage phiX174. In addition, patients have an impaired skin barrier, which increases the likelihood of cutaneous infections and bloodstream bacterial infections (most commonly with *S. aureus*) (192).

Clinically, the HIES symptomatic triad of atopic dermatitis and recurrent infections, eosinophilia, as well as elevated IgE is present in Comel-Netherton patients. The definitive non-genetic diagnostic signs for Comel-Netherton syndrome are the abnormalities in the hair shaft in combination with the other syndromic features. **Table 1** shows the summary of Hyper IgE syndromes describing the primary causative gene, their mode of inheritance and further details. Multiple case reports have documented significant clinical improvement in both adults and children received dupilumab treatment (193–196). Treatment with omalizumab reduced skin and mucosal symptoms in a 20-year-old Comel-Netherton patient (197). A Notable skin improvement was shown in a young adult with Comel-Netherton after omalizumab therapy (198). Promising results were achieved using monoclonal antibodies targeting IL-17, such as ixekizumab and secukinumab, along with TNF-α inhibitors like infliximab and anakinra, for managing inflammatory skin lesions in Comel-Netherton (199–203).

**Table 1:** Summary of Hyper IgE syndromes describing the primary causative gene, their mode of inheritance.

Syndrome	Gene		Symptoms				
		Immunological Features	Infectious Susceptibilities	Atopic/Allergic Manifestations	Musculoskeletal/Connective Tissue	Th17 cell levels*	Other Clinical Features
AD (DN effect) Job’s Syndrome	*STAT3*	Hyper IgE, High Eosinophils	Skin infections, Mucocutaneous candidiasis, Pulmonary infections	Severe eczema	Minimal trauma fractures, Retained primary teeth, connective tissue (like: vascular tortuosity and aneurysm) and Skeletal tissue abnormalities.	reduced (215)	Malignancy
AR ZNF341 Deficiency	*ZNF341*	Hyper IgE, High Eosinophils	Skin infections, Mucocutaneous candidiasis	Severe eczema	Minimal trauma fractures, Retained primary teeth, Skeletal and connective tissue abnormalities.	normal or reduced (166)	
AR GP130 deficiency	*IL6ST*	Hyper IgE, High Eosinophils	Skin infections, Mucocutaneous candidiasis, Pulmonary infections	Severe eczema	Minimal trauma fractures, Retained primary teeth, Skeletal and connective tissue abnormalities.	normal (17)	
AR IL-6 receptor deficiency	*IL6R*	Hyper IgE, High Eosinophils	Skin infections, Mucocutaneous candidiasis, Pulmonary infections	Severe eczema	Minimal trauma fractures, Retained primary teeth, Skeletal and connective tissue abnormalities.	normal or reduced (77)	
AR *DOCK8* deficiency	*DOCK8*	Hyper IgE, High Eosinophils	Skin infections, Mucocutaneous candidiasis, Warts, Herpes viridae infections	Severe eczema, [Severe] food allergies		reduced (216)	Malignancy
AR PGM3 deficiency	*PGM3*	Hyper IgE, High Eosinophils	Herpes viridae infections,	[Severe] food allergies, Asthma	Skeletal and connective tissue abnormalities.	reduced (176)	Malignancy, Neurocognitive delays
AD (DN effect) CARD11 deficiency	*CARD11*	Hyper IgE, High Eosinophils	Molluscum, Pulmonary infections	Severe eczema, [Severe] food allergies, Asthma.		normal (121)	Malignancy, Bamboo hair
AR Comel-Netherton Syndrome	*SPINK5*	Hyper IgE, High Eosinophils		Severe eczema, [Severe] food allergies, Allergic rhinitis, Asthma		ND	Enteropathy
AD GOF STAT6 syndrome	*STAT6*	Hyper IgE, High Eosinophils	Skin infections, Molluscum, Pulmonary infections	Severe eczema, [Severe] food allergies, Allergic rhinitis, Eosinophilic GI disease, Asthma.	Skeletal and connective tissue abnormalities	normal or reduced (217)	Malignancy, Enteropathy
AD Loeys-Dietz syndrome	*TGFBR*	Hyper IgE		Severe eczema	connective (like: thoracic aortic aneurysm) and Skeletal tissue abnormalities.	ND (218)	EBV-associated malignancy
AD ERBIN Deficiency	*ERBIN*	Hyper IgE, High Eosinophils		Severe eczema, Eosinophilic GI disease	Skeletal and connective tissue abnormalities.	normal (108)	EBV-associated malignancy

AD, Autosomal dominant; AR, Autosomal recessive, and the associated symptoms. *Th17 cell levels do not necessarily indicate normal differentiation. ND, No Data.

### Other primary immunodeficiency disorders with elevated serum IgE levels

High IgE levels are a feature of numerous other PIDs falling under the combined immunodeficiency category. These conditions encompass Omenn syndrome, DiGeorge syndrome, and Wiskott-Aldrich syndrome, all distinguished by significant decreases in T-cell counts and profound deficiencies in T-cell functionality (54, 204–206). The clonal expansion of specific T and B cells, reduced TCR affinity and signaling, or an imbalance in the development of Th1/Th2 cells, could all contribute to elevated levels of serum IgE (207). Immune dysregulation, polyendocrinopathy, enteropathy X-linked (IPEX) syndrome, caused by *FOXP3* LOF mutations, also feature elevated serum IgE levels and an absence of Treg cells, indicating that the absence of Treg cells by itself is adequate to cause increased serum IgE levels in affected patients. Further, additional skin barrier syndromes, which may or may not lead to IgE elevation, and may or may not lead to increased infection (certainly not beyond the skin) include SAM syndrome, complete filaggrin deficiency, epidermolysis bullosa. However, in these cases the elevation in IgE levels is thought to occur due to a standard atopic response caused by an increased degree of environmental allergen exposure, as opposed to a direct genetic deficiency in the immune tolerogenic pathway that underlies HIES cases. To effectively diagnose HIES, it is also essential to rule out these alternative conditions which can be carried out through genetic screening approaches (1) or with clinical diagnosis as in the cases of alternative IEIs with high IgE such as Omenn syndrome and IPEX which are very distinct and do not overlap with the traditional HIES.

## Conclusions

HIES is defined as a complex PID, distinguished by hyper-elevated serum IgE levels, recurrent infections, and atopic dermatitis (50). Genetic categorization reveals that HIES can be inherited in either an AR or AD manner or may arise from *de novo* spontaneous genetic changes. While the majority of patients with AD-HIES are linked to mutations in the *STAT3* gene, most instances of AR-HIES are connected to mutations in *DOCK8* (6). However, recent research has identified other genetic disorders linked to HIES, including mutations in *IL6ST, PGM3, ERBIN, ZNF341, IL6R, SPINK5, TGFBR1, TGFBR2, STAT6* and *CARD11*, expanding our knowledge of the genetic basis for this syndrome (133, 141, 208).

Atopic conditions, such as food allergies, are a significant feature of hyper-IgE syndromes (HIES), with their presentation varying based on the underlying genetic mutation.Patients with DOCK8, STAT3, or atopic dermatitis exhibit distinct sensitization patterns (209), with allergic symptoms and skin prick test outcomes strongly aligning with specific IgE levels in DOCK8 and atopic dermatitis cases, aiding dietary management decisions (210). A global study of 136 DOCK8 cases revealed that more than 70% experienced allergy-related symptoms, including food-induced anaphylaxis in 16%, as well as drug allergies, asthma, and rhinitis (163) Food allergies in DOCK8-HIES patients can persist even after HSCT, despite achieving full donor chimerism (211) PGM3-HIES patients often have multiple allergies (168), whilst WAS patients and murine models frequently develop IgE-mediated reactions to environmental and food allergens (212). STAT3-HIES patients have specific IgE levels and skin prick test outcomes similar to those of healthy individuals, despite their extremely high total IgE levels (209), and they experience higher rates of lifetime prevalence and severity of food allergies than healthy controls but lower rates compared to atopic controls with similar IgE levels. This phenomenon may be due to the severe impairment of mast cell degranulation associated with STAT3 loss-of-function mutations (213). Understanding these genetic distinctions is crucial for developing tailored management strategies for food allergies in HIES patients.

Understanding the distinct molecular mechanisms of AD-HIES and AR-HIES is essential for precise diagnosis, management, and the establishment of targeted therapeutic strategies. Although HSCT has demonstrated promise in restoring immune function and decreasing IgE levels in affected individuals, additional research is required to clarify the specific biological mechanisms driving the clinical variations observed between the AD and AR inherited forms of HIES. HSCT presents challenges, since donors may not always be readily available, including the reactivation of viral infections, the potential for graft-versus-host disease, and donor chimerism. Hence, innovative and promising approaches to treating DOCK8 deficiency include adaptive cellular therapies and gene therapy. However, these methods are still being investigated (46, 61, 214). Ultimately, continued investigation into the genetic and molecular basis of HIES will facilitate the development of more efficient therapeutic interventions and enhance the outcomes for individuals affected by these rare immunodeficiency disorders.
